# From activity to obesity: understanding gender and type of school divide among Saudi adolescents

**DOI:** 10.3389/fpubh.2025.1552243

**Published:** 2025-02-27

**Authors:** Mohamed Ahmed Said, Mohammed Shaab Alibrahim

**Affiliations:** Department of Physical Education, College of Education, King Faisal University, Al-Ahsa, Saudi Arabia

**Keywords:** obesity, physical activity, sedentary behavior, school type, adolescents

## Abstract

**Background:**

Obesity has emerged as a significant predictor of the nationwide burden of non-communicable diseases in Saudi Arabia.

**Objectives:**

This study explores patterns in body mass index (BMI), physical activity (PA), and sedentary behavior (SB) among Saudi adolescents, focusing on variations by gender and school type (public vs. private). It identifies key factors affecting BMI.

**Methods:**

A total of 2,815 students (53.64% male, aged 15.21 ± 1.55 years) participated. Body weight and composition were determined by bioelectrical impedance, while PA levels and SB scores were assessed through validated questionnaires. Two models were created, one with overall SB and the other with specific SBs.

**Results:**

Among participants, 28.4% were overweight/obese, with 17.2% classified as obese. Obesity prevalence was higher in boys (26.9%) compared to girls (6.0%). Overweight prevalence was slightly lower in private schools (9.9%) than in public schools (11.8%), while obesity rates were similar (17.3% vs. 17.2%). A significant association between BMI and school type was identified solely in girls (Model 1: *p* = 0.004, ES = 0.109; Model 2: *p* = 0.012, ES = 0.096). Age was positively associated with BMI (Model 1: *p <* 0.004, ES = 0.025; Model 2: *p <* 0.001, ES = 0.019), as were SB scores (*p <* 0.001, ES = 0.17). Conversely, PA levels exhibited a negative correlation with BMI (Model 1: *p <* 0.001, effect size = −0.104; Model 2: *p <* 0.001, effect size = −0.106). Polynomial analysis revealed a cubic relationship between BMI and PA across all groups, though with low effect sizes.

**Conclusion:**

Gender, age, PA, and SB explained a small portion of BMI variance. Future research should investigate mechanisms underlying these non-linear trends and explore additional confounding variables.

## Introduction

In recent decades, the prevalence of obesity has emerged as a significant predictor of the global burden of non-communicable diseases (1). Obesity is associated with diverse medical issues, including type 2 diabetes mellitus, high cholesterol, high blood pressure, lung disease, rheumatoid arthritis, sleep apnea, colon disease, and thyroid illness (1–3). It is estimated that half of all cases of childhood obesity progress into adolescent obesity, with nearly 80% continuing into adulthood (4). The WHO has predicted that approximately 20% of the global population will be obese by 2050. Therefore, addressing and controlling childhood obesity is crucial for promoting optimal health and minimizing the increased susceptibility to chronic diseases in adulthood (5).

Numerous studies have established a positive association between physical activity (PA) and various parameters, including body composition (6), physical fitness (7), cardiometabolic biomarkers (8, 9), academic performance (10, 11), and psychological well-being (12), in children and adolescents. The WHO’s guidelines on PA and sedentary behavior [SB; (13)] indicate that individuals aged 5–17 years should engage in at least 60 min of daily PA, including aerobic exercises and activities that promote muscular and bone development. The overall magnitude of PA is more influential than factors such as frequency, duration, and variety of activities (e.g., aerobic exercises, resistance training, and bone-strengthening exercises) concerning health benefits for adolescents (14).

Despite the well-documented benefits of PA, most Saudi adolescents fail to meet recommended guidelines. Studies indicate that 82.4% of school-aged Saudi adolescents do not achieve the recommended 60 min of moderate-to-vigorous-intensity PA per day, while 59% fail to engage in sufficient vigorous-intensity activity at least 3 days per week (15, 16). This issue is exacerbated by rapid societal and infrastructural changes in Saudi Arabia, particularly over the past 5 years, which underscore the need for deeper research into the opportunities and barriers for PA, especially among females.

Barriers to PA among adolescents are multifaceted, encompassing individual, social, and environmental factors. Schools play a critical role in shaping PA levels; however, financial constraints and inadequate resources can limit the availability of quality PA programs (17, 18). Social and institutional systems within schools also have a profound influence, with the school atmosphere either encouraging or discouraging PA (19). Ecological behavior models suggest that external factors, such as the availability of supportive environments, are crucial in influencing individual choices (20).

Cultural norms and traditions play a significant role in influencing PA levels, particularly for females, where societal attitudes and gender-based restrictions often act as barriers (21). Although initiatives under Saudi Arabia’s Vision 2030 aim to improve women’s access to sports—such as introducing sports in females’ schools, licensing women-only gyms, and organizing female sports competitions—participation rates for women remain lower than those for men (22, 23). Environmental challenges, such as the lack of safe, accessible public spaces and the region’s harsh Saharan climate, further discouraging outdoor activities (22–24).

Understanding the impact of school type (public vs. private) on adolescents’ PA and SB is essential, as these factors directly affect BMI and obesity rates. Public and private schools differ in terms of resources, infrastructure, and emphasis on physical education, all of which influence students’ opportunities for PA (16). Additionally, cultural and socioeconomic factors, including societal norms and limited access to recreational facilities—particularly for women—may exacerbate disparities (22, 24). Exploring these differences by school type and gender provides valuable insights into BMI trends and key influencing factors, which can inform targeted interventions to reduce obesity.

Economic disparities also influence PA engagement, as individuals from higher-income households often have access to private gyms and recreational facilities, while those from lower-income households face limited opportunities (25). Furthermore, sedentary lifestyles, characterized by increased screen time and reliance on motorized transportation, restrict PA levels among adolescents (26). Addressing these barriers requires comprehensive public health interventions, including targeted policies, improved school programs, and culturally sensitive campaigns, to promote active lifestyles across all demographics.

Many studies indicate that a significant proportion of children spend most of their time engaged in SBs (27, 28). SB has notably increased over the past decade (29). SBs have been increasingly recognized in health promotion initiatives due to their potential correlation with adverse cardiometabolic and mental health outcomes, decreased bone mineral content, and higher rates of overweight and obesity among individuals aged <18 years (30, 31). Tremblay et al. (32) explored the concept of sedentary time and its relationship with SB, including the duration and context in which individuals engage in such behaviors. Temmel and Rhodes (33) have identified several unique SBs in children, particularly during their leisure time, which must be distinguished for effective measurement.

Screen time is a commonly measured factor in studies on SB. According to Tremblay et al. (32), “screen time” refers to activities involving screens, such as watching television (TV), using computers, and playing video games. Biddle et al. (34) indicated that these activities account for approximately one-third of total sedentary time. It is crucial to understand that sedentary habits and screen-based activities are distinct behaviors requiring individual examination (35). To enhance the effectiveness of intervention programs aimed at reducing screen time, it is vital to comprehensively understand the complex characteristics associated with screen-related behaviors in children and adolescents. This understanding should consider various scenarios, including distinct cultural environments and individual circumstances (36). By identifying and analyzing multiple aspects, such as gender, age, cultural contexts, and climatic conditions, researchers can gain insights into the potential influences on these behaviors.

This study aimed to uncover patterns in BMI and obesity, as well as PA and SB levels, among Saudi adolescents, focusing on variations by school type (public vs. private) and gender. It also aimed to identify the key factors affecting BMI, examining the influences of school type, gender, age, PA, and general and specific SB, including watching TV, laptop use, video gaming, and smartphone use. These aims will enhance comprehension of the complex interactions influencing BMI variability in various educational settings, providing insights to inform targeted health interventions for adolescents in Al-Ahsa Governorate, Saudi Arabia.

## Methods

### Study design

This study used a quantitative research approach, including surveys and bioelectrical impedance analysis, to investigate patterns in BMI, PA levels, and SB scores among Saudi adolescents. The study also looked at differences based on school type (public versus private) and gender. Data collection occurred between April and June 2023, and the study methodology was approved by the Deanship of Scientific Research Ethics Committee at King Faisal University (KFU-REC-2022-OCT-ETHICS202).

### Participants

Participants were selected through a multistage cluster random sampling method, choosing 20 public and 10 private schools from the pool of middle and high schools in Al-Ahsa Governorate, situated in the Eastern Province of Saudi Arabia. The 30 selected schools were equally divided between males’ and females’ schools, with six from each geographic area (East, West, Center, North, and South). Geographic regions were defined based on the distribution of schools within Al-Ahsa Governorate’s physical layout. To ensure the selected regions accurately represented the diversity of the student population in the study area, the geographic distribution was cross-referenced with current census data and reports from the Saudi Ministry of Education—General Education Statistics (37). This study then used a random selection process to choose 1–2 classes at each grade level within each school. The chosen classes typically had a size of 15–30 students. Any students who met one or more exclusion criteria were excluded from this study, while all other students were encouraged to participate. The exclusion criteria were as follows: (1) not scheduled for physical education (PE) classes, (2) having a disability, (3) having an acute or chronic medical condition, (4) failure to complete one or more study phases, and (5) no documented parental agreement for involvement in this study. Of the 2,899 affirmative replies obtained from parents, 84 students were removed due to morbid obesity (*n* = 6), inability to complete all stages (*n* = 4), or lack of interest (*n* = 74). Therefore, 2,815 students successfully completed all phases of this study. Before the study commenced, all participants and their parents were provided with comprehensive information about its research protocol. In accordance with ethical guidelines, students aged ≥16 years and the parents of students aged <16 years were required to provide written informed consent before their enrollment in this study.

### Protocol

The participants were evaluated during their regular physical education (PE) classes. They filled out a pre-determined questionnaire and were measured for several anthropometric measures, including height, body weight, and body composition. The questionnaire assessed individuals’ PA level and SBs score. It consisted of three unique components and took about 25 min to complete. The first component collected participants’ demographic information: age, grade, and gender. The second component was the Arabic version of the Physical Activity Questionnaire for Adolescents (PAQ-A) developed by Kowalski et al. (38), which is appropriate for students in grades 9–12 (approximately aged 14–20 years). The third component was the NSW Schools PA and Nutrition Survey (SPANS 2010) developed by Hardy et al. (39), which contains questions about SBs.

Anthropometric measurements were conducted by PE instructors in controlled, air-conditioned classrooms to ensure consistency. Data was manually recorded on pre-prepared cards, with participants organized according to the order of their names on the class register. The questionnaire was digitized and converted into an online survey via Google Drive, allowing participants to access it using school-provided computers. Participants were required to specify their class and their designated number from the class register for identification reasons. To preserve anonymity, all data were associated solely with student numbers on the class register, and no identifiable personal information was gathered. For participants who encountered difficulties or confusion with any part of the questionnaire, a researcher was available to provide clarification and assistance.

### Outcomes

#### Anthropometry

Height was assessed using a stadiometer (Holtain, Crymych, Wales, UK). Body weight, BMI, and percentage body fat (PBF) were evaluated using a bioelectrical impedance body composition monitor (Omron BF508, Kyoto, Japan). This method has been validated in similar populations by comparing it with gold-standard techniques, such as dual-energy X-ray absorptiometry (DXA) and multi-frequency bioelectrical impedance spectroscopy (40). All measures were performed in accordance with the manufacturer’s specifications, ensuring participants wore minimal clothing, had clean feet, and maintained uniform hydration levels. Height, age, and sex were manually entered into the bioelectrical impedance analyzer, while weight, body fat percentage, and BMI were registered.

A run-in phase was organized during the week preceding the tests to verify the methodology and guarantee measurement precision and accuracy. A visit to each school was arranged to familiarize educators with the evaluation technique, equipment, and procedures. At the end of the run-in phase, each instructor was mandated to administer each test a minimum of five times with minimal errors. An “error” is defined as any variation from the established methodology, including the use of improper equipment or the input of erroneous data. Students participating in this phase were excluded from the main study.

Fat mass (FM) was calculated by multiplying body weight (in kg) by PBF, and fat-free mass (FFM) was calculated by subtracting FM from body weight. Participants were categorized into four groups based on the World Health Organization growth reference data for individuals aged 5–19 years (41): underweight (UW; BMI-for-age < 5th percentile), normal weight (NW; 5th percentile ≤BMI-for-age < 85th percentile), overweight (OW; 85th percentile ≤BMI-for-age < 95th percentile), and obesity (OB; BMI-for-age ≥ 95th percentile).

#### Physical activity level

PA levels were assessed using the PAQ-A, translated and adapted for the Saudi context by our research team (42). The findings indicated that the Arabic version of the PAQ-A exhibited good psychometric properties. The Kaiser-Meyer-Olkin measure was 0.697, and Bartlett’s test produced a significant result (*p <* 0.001). Factor analysis identified three components that explained 55.762% of the variance, with item loadings ranging from 0.591 to 0.811. Cronbach’s alpha was 0.69, indicating acceptable reliability, with all items demonstrating adjusted item-total correlations exceeding 0.30.

This self-administered questionnaire covers 7 days and assesses engagement in PA during lunch breaks, after school, on holidays, and within PE programs. It comprises nine items, with the first eight evaluated on a five-point Likert scale from 1 to 5, excluding item 9. A mean composite score was calculated for items 1–8, with a score of 1 indicating low PA and 5 indicating high PA. Participants were grouped into five categories based on their mean composite scores: extremely inactive (<1.8), inactive (1.8–<2.60), moderately active (2.60–<3.4), active (3.4–<4.2), and very active (≥4.2).

#### Sedentary behavior

SBs were evaluated utilizing the SPANS (2010), which was also translated and modified for the Saudi context by our research team (43). The assessment comprises five items that evaluate SBs during weekdays and weekends, including the use of digital devices and the internet (e.g., tablet, computer, or smartphone), watching television, films, or online content, and engaging in video games (on a computer, gaming console, smartphone, or tablet). The reliability of the translated SPANS was confirmed with a satisfactory Cronbach’s alpha (*α* = 0.78). Participants evaluated their SB using a scale from 0 to 5, where 0 indicates no activity, 1 represents less than 30 min per day, 2 corresponds to 30 min to 1 h per day, 3 signifies 1 to 2 h per day, 4 denotes 2 to 4 h per day, and 5 reflects more than 4 h per day. A mean SPANS score, which ranges from 0 to 5, was calculated for each participant, with higher scores reflecting increased SB. Participants were classified into six levels of SB based on interval width: very low SB (<0.83), low SB (0.83–<1.83), moderately low SB (1.83–<2.66), moderately high SB (2.66–<3.49), high SB (3.49–<4.32), and very high SB (≥4.32).

In Saudi Arabia, weekdays are Sunday through Thursday, and weekend days are Friday and Saturday. The school day spans from the start of the academic day when the school bell rings, usually around 7:00 am, to its conclusion around 12:30 pm. After-school time typically lasts 120 min, covering the time students take to return home and their activities once they arrive home. The “evening” period refers to the 3 h from 3:00 pm to 8:00 pm, concluding after the end of after-school activities.

### Statistical analysis

Statistical analyses were performed using SPSS Statistics (version 26; IBM Corp., Armonk, NY, USA). A *p <* 0.05 was considered statistically significant. Participants’ characteristics are summarized using descriptive statistics, including mean ± standard deviation (SD). The normality of variable distributions was assessed by examining histograms and calculating absolute skewness and kurtosis values, with thresholds of absolute skewness >2 or kurtosis >7 used to indicate significant non-normality. Differences by gender and school type were examined using Student’s *t*-test. Cohen’s *d* was used to quantify effect sizes (ES) for differences in height, body weight, body composition, PA levels, and SB scores, which were classified as small (0.2), medium (0.5), or large (0.8).

The relationships between BMI and PA levels were examined using linear, quadratic, and cubic models, guided by existing evidence and visual assessment of scatterplots, which suggested possible non-linear trends. Model fit was evaluated using the coefficient of determination (*R*^2^), with the cubic model showing the highest explanatory power. However, due to the multicollinearity and heteroscedasticity of some predictors, a generalized linear model (GLM) was used for robust estimation. Two models were constructed to account for potential confounding factors: one included the overall SB score, and the other incorporated specific SBs. These approaches ensured reliable analysis of predictors’ effects on BMI. Effect sizes, expressed as standardized coefficients, were calculated for both continuous and categorical predictors using the formula: ES = *β* × (SD of the predictor / SD of BMI), avec β the unstandardized coefficient. For categorical predictors, binary coding was applied, with SD of the dummy-coded variable approximated as √0.5. For continuous predictors, their observed SDs were used directly. These standardized coefficients provide a consistent scale for comparing the relative impact of different predictor variables on BMI, aiding in the interpretation of effect sizes (44, 45).

## Results

### Participants’ characteristics

This study involved 2,815 students who completed all experiment steps, of which 1,876 were from public schools and 939 were from private schools. In addition, 1,510 were male (53.64%), of whom 946 attended public schools and 564 attended private schools, and 1,305 were female (46.36%), of whom 930 attended public schools and 375 attended private schools. The participants’ ages ranged from 13 to 18 years, with a mean of 15.21 ± 1.55 years (Table 1).

**Table 1:** Participants’ age, anthropometric parameters, composite PAQ-A scores, and mean SB scores.

Variable	*N*	Mean	SD	Min	Max	Skewness	Kurtosis
Age (years)	2,815	15.21	1.55	13.00	18.00	0.27	−1.04
Height (cm)	2,815	151.63	12.19	82.00	190.00	−0.22	0.43
Weight (kg)	2,815	46.13	13.57	18.00	122.50	1.10	2.22
BMI (kg/m^2^)	2,815	19.85	4.50	6.00	45.94	1.29	2.57
PBF (%)	2,815	19.40	8.30	2.95	54.70	0.86	1.23
FM (kg)	2,815	9.66	6.62	0.62	50.00	2.01	6.50
FFM (kg)	2,815	36.48	8.83	16.09	87.00	1.19	2.50
PA level	2,815	2.33	0.75	1.00	4.04	0.39	0.22
SB score	2,815	2.77	0.89	0.00	5.00	0.08	0.12
Watching TV score	2,815	3.03	1.41	0.00	5.00	−0.25	−0.69
Laptop use score	2,815	2.65	1.57	0.00	5.00	0.03	−1.09
Playing video-games score	2,815	2.18	1.59	0.00	5.00	0.22	−1.08
Smartphone use score	2,815	3.29	1.42	0.00	5.00	−0.36	−0.86

BMI, body mass index; FM, fat mass; FFM, fat-free mass; PAQ-A, Physical Activity Questionnaire for Adolescents; PBF, percentage body fat; SB, sedentary behavior; SD, standard deviation.

Among participants, 28.4% were classified as OW or OB, with 17.2% classified as OB. Among males, 41.1% were OW or OB, with 26.9% classified as OB, while among females, only 14.9% were OW or OB, with 6.0% classified as OB. Females exhibited a higher prevalence of NW (71.0% vs. 48.2%) and UW (14.1% vs. 11.7%) than males. Those at private schools exhibited a slightly higher prevalence of NW (60.9% vs. 57.7%) and a lower prevalence of OW (9.9% vs. 11.8%) than those at public schools, with a nearly identical prevalence of OB (17.3% vs. 17.2%).

The mean composite PAQ-A score was 2.33 ± 0.75, indicating that most participants were inactive (1.8–<2.6). The mean SB score was 2.77 ± 0.89, indicating that most participants exhibited moderately high SB (2.6–<3.4). Among the specific SBs, smartphone use (3.29 ± 1.42) and watching TV (3.03 ± 1.41) were the most common, whereas laptop usage (2.65 ± 1.57) and playing video games (2.18 ± 1.59) were the least common, corresponding to moderately high and moderately low SB classifications, respectively.

Regarding BMI categories, participants who were UW were more physically active than others. Specifically, they were more active compared to those who were NW (*ρ* < 0.001, Cohen’s d = 0.2599), OW (*ρ* = 0.035, Cohen’s d = 0.2323), and OB (*ρ* < 0.001, Cohen’s d = 0.4927). They were also less sedentary compared to OW participants (*ρ* = 0.004, Cohen’s d = 0.2463) and OB participants (*ρ* < 0.001, Cohen’s d = 0.3103).

**Figure 1:**
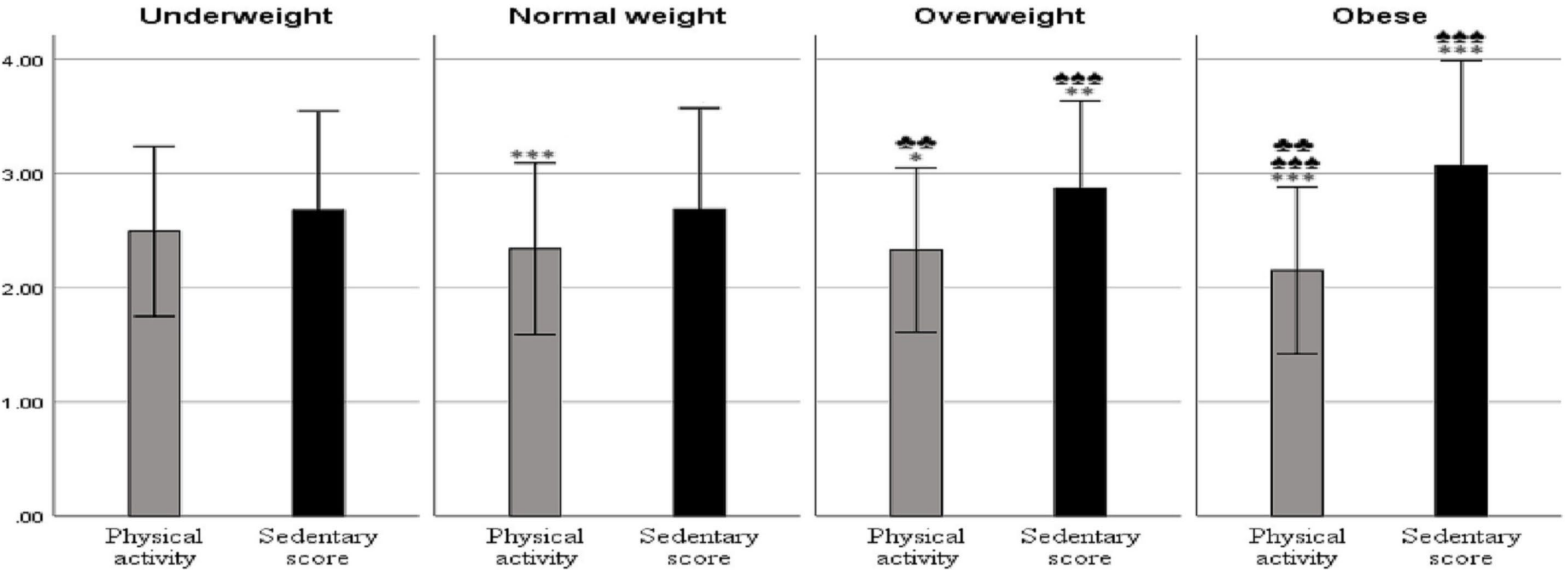
Physical activity levels and sedentary behavior scores by BMI categories. Results are presented as mean ± 1 SD; *p < 0.05, **p < 0.01, ***p < 0.001 relative to the underweight group; ♠*p <* 0.05, ♠♠*p <* 0.01, ♠♠♠*p <* 0.001 relative to the normal weight group; ♣*p <* 0.05, ♣♣*p <* 0.01, ♣♣♣*p <* 0.001 relative to the overweight group.

Moreover, participants categorized as OB were less physically active than their OW counterparts (*ρ* = 0.002, Cohen’s d = 0.2459) and NW counterparts (*ρ* < 0.001, Cohen’s d = 0.2253). Additionally, OB participants were more sedentary compared to those in the NW category (*ρ* < 0.001, Cohen’s d = 0.2834). No significant differences were observed in PA levels between NW and OW participants or in SB scores between OW and OB participants (Figure 1).

### Participants’ characteristics according to school type and gender

Participants from private schools differed significantly from those from public schools in height (*p <* 0.001; Cohen’s d = 0.936), weight (*p <* 0.001; Cohen’s d = 0.239), and PBF (*p <* 0.001; Cohen’s d = 0.382; Table 2). Regarding gender, males attending public schools were taller and heavier than their counterparts in private schools but had lower PBF (*p <* 0.001for all variables, with Cohen’s d = 0.857 for height, 0.402 for weight, 0.134 for BMI, 0.304 for PBF, and 0.538 for FFM). In contrast, females attending public schools were shorter than those attending private schools (*p <* 0.001; Cohen’s d = 0.561). Although their body weights were comparable, females in public schools had significantly higher PBF (*p <* 0.001; Cohen’s d = 0.987). Table 2 provides a detailed comparison of participants’ anthropometric measurements based on gender, school type, and their interaction.

**Table 2:** Participants’ ages and anthropometric parameters by gender and school type.

Variable	School type	Gender	*N*	Mean	SD	Comparison		
						*t*	*p*	Cohen’s *d*
Age (years)	Public	Male	946	14.75	1.55	−16.560	<0.001	0.778
		Female	930	15.86	1.29			
		Total	1876	15.25	1.54			
	Private	Male	564	14.36	1.42	−18.014	<0.001	1.174
		Female	375	15.93	1.25			
		Total	939	15.13	1.55			
	Public vs. Private	Male				4.897	<0.001	0.262
		Female				0.89	0.343	0.055
		Total				1.979	0.048	0.078
Height (cm)	Public	Male	946	150.96	15.34	−8.092	<0.001	0.374
		Female	930	155.39	6.84			
		Total	1876	155.93	1.64			
	Private	Male	564	141.78	9.39	−30.801	<0.001	1.422
		Female	375	158.84	6.37			
		Total	939	144.43	0.97			
	Public vs. Private	Male				12.860	<0.001	0.857
		Female				−8.420	<0.001	0.561
		Total				23.330	<0.001	0.936
Weight (kg)	Public	Male	946	46.64	17.30	−1.478	0.140	0.068
		Female	930	47.62	10.89			
		Total	1876	47.125	14.02			
	Private	Male	564	41.58	12.84	−8.952	<0.001	0.413
		Female	375	48.03	6.69			
		Total	939	44.152	11.263			
	Public vs. Private	Male				6.030	<0.001	0.402
		Female				−0.67	0.503	0.045
		Total				5.985	<0.001	0.239
BMI (kg/m^2^)	Public	Male	946	19.97	4.95	1.479	0.139	0.068
		Female	930	19.67	4.03			
		Total	1876	19.821	4.518			
	Private	Male	564	20.52	5.38	5.121	<0.001	0.236
		Female	375	19.02	2.25			
		Total	939	19.922	4.462			
	Public vs. Private	Male				−2.010	0.044	0.134
		Female				2.930	0.003	0.195
		Total				−0.561	0.575	0.022
PBF (%)	Public	Male	946	14.21	5.80	−38.968	<0.001	1.800
		Female	930	26.50	7.71			
		Total	1876	20.301	9.173			
	Private	Male	564	15.68	6.47	−13.529	<0.001	0.625
		Female	375	20.45	2.72			
		Total	939	17.586	5.788			
	Public vs. Private	Male				−4.560	<0.001	0.304
		Female				14.810	<0.001	0.987
		Total				9.567	<0.001	0.382
FM (kg)	Public	Male	946	7.35	<0.001	−19.633	<0.001	0.907
		Female	930	13.33	7.40			
		Total	1876	10.315	7.227			
	Private	Male	564	7.25	5.79	−8.540	<0.001	0.394
		Female	375	9.97	2.66			
		Total	939	8.336	4.973			
	Public vs. Private	Male				0.340	0.732	0.023
		Female				8.560	<0.001	0.570
		Total				8.504	<0.001	0.340
FFM (kg)	Public	Male	946	39.29	<0.001	11.640	<0.001	0.538
		Female	930	34.29	4.45			
		Total	1876	36.811	9.678			
	Private	Male	564	34.33	7.70	−8.559	<0.001	0.395
		Female	375	38.05	4.21			
		Total	939	35.816	6.778			
	Public vs. Private	Male				8.560	<0.001	0.570
		Female				−14.040	<0.001	0.936
		Total				3.165	0.002	0.125

BMI, body mass index; FM, fat mass; FFM, fat-free mass; PBF, percentage body fat; SD, standard deviation.

Participants attending private schools demonstrated higher levels of PA (*p <* 0.001; Cohen’s d = 0.323) and engaged in more SBs (*p* = 0.013; Cohen’s d = 0.099) compared to those from public schools (Table 3). They also reported higher levels of watching TV (*p <* 0.001; Cohen’s d = 1.149), laptop use (*p <* 0.001; Cohen’s d = 0.396), and video games playing (*p <* 0.001; Cohen’s d = 0.160). However, participants from private schools exhibited lower levels of smartphone use compared to their public-school counterparts (*p <* 0.001; Cohen’s d = 0.132).

**Table 3:** Participants composite PAQ-A and SB scores by gender and school type.

Variable	School type	Gender	*N*	Mean	SD	*t*	*p*	Cohen’s *d*
PA level	Public	Male	946	2.406	0.750	8.801	<0.001	0.406
		Female	930	2.093	0.790			
		Total	1876	2.251	0.785			
	Private	Male	564	2.600	0.680	7.639	<0.001	0.353
		Female	375	2.280	0.550			
		Total	939	2.476	0.650			
	Public vs. Private	Male				−5.140	<0.001	0.343
		Female				−4.270	<0.001	0.284
		Total				−8.069	<0.001	0.323
SB score	Public	Male	946	2.887	0.875	7.651	<0.001	0.353
		Female	930	2.593	0.820			
		Total	1876	2.741	0.846			
	Private	Male	564	2.790	0.950	−1.846	0.065	0.085
		Female	375	2.900	0.970			
		Total	939	2.833	0.960			
	Public vs. Private	Male				2.150	0.032	0.143
		Female				−5.880	<0.001	0.392
		Total				−2.474	0.013	0.099
Watching TV scores	Public	Male	946	2.730	1.470	−7.040	<0.001	0.325
		Female	930	3.190	1.380			
		Total	1876	2.961	1.444			
	Private	Male	564	2.960	1.370	−5.788	<0.001	0.267
		Female	375	3.470	1.190			
		Total	939	3.165	1.324			
	Public vs. Private	Male				−3.060	0.002	0.204
		Female				−3.340	<0.001	0.223
		Total				−3.737	<0.001	0.149
Laptop use score	Public	Male	946	2.590	1.500	3.425	<0.001	0.158
		Female	930	2.340	1.650			
		Total	1876	2.464	1.585			
	Private	Male	564	2.780	1.410	−7.238	<0.001	0.334
		Female	375	3.480	1.470			
		Total	939	3.062	1.472			
	Public vs. Private	Male				−2.510	0.012	0.167
		Female				−11.66	<0.001	0.777
		Total				−9.904	<0.001	0.396
Playing video-games score	Public	Male	946	2.930	1.530	24.235	<0.001	1.119
		Female	930	1.250	1.470			
		Total	1876	2.101	1.721			
	Private	Male	564	2.750	1.320	13.200	<0.001	0.610
		Female	375	1.710	0.940			
		Total	939	2.330	1.289			
	Public vs. Private	Male				2.400	0.017	0.160
		Female				−5.540	<0.001	0.369
		Total				−3.992	<0.001	0.160
Smartphone use score	Public	Male	946	3.290	1.450	−4.342	<0.001	0.201
		Female	930	3.580	1.340			
		Total	1876	3.434	1.406			
	Private	Male	564	2.710	1.380	−7.778	<0.001	0.359
		Female	375	3.410	1.330			
		Total	939	2.993	1.405			
	Public vs. Private	Male				7.669	<0.001	0.511
		Female				1.976	0.048	0.132
		Total				7.858	<0.001	0.314

PA, Physical Activity; SB, sedentary behavior.

Across both school types, males tended to be more active and sedentary than females, except for males attending private schools, who were equally sedentary as females (*p* = 0.032 for public vs. private school males; Cohen’s d = 0.343; *p <* 0.001 for all other comparisons, with Cohen’s d = 0.406 and 0.353 for public schools and 0.353 for private schools). Females attending private schools reported significantly higher levels of TV watching and laptop use compared to males in the same schools (*p <* 0.001 for both; Cohen’s d = 0.267 and 0.334, respectively) and to females attending public schools (*p <* 0.001 for both; Cohen’s d = 0.223 and 0.777, respectively). In contrast, males attending public schools reported the lowest levels of TV watching and laptop use compared to their peers attending private schools (*p* = 0.002 and 0.012; Cohen’s d = 0.204 and 0.167, respectively).

Males generally played video games more frequently than females (*p <* 0.001 for all comparisons, with Cohen’s d = 0.610 for private schools and Cohen’s d = 1.119 for public schools). Video game playing also differed significantly between public and private schools for both males (*p* = 0.017; Cohen’s d = 0.160) and females (*p <* 0.001; Cohen’s d = 0.369).

Finally, students attending public schools used smartphones more frequently than those in private schools (*p* = 0.048 for females, Cohen’s d = 0.132; *p <* 0.001 for all the rest comparisons; Cohen’s d = 0.314 overall; Cohen’s d = 0.511 for males), with significantly greater smartphone use reported among females compared to males in both type of schools (*p <* 0.001 for all comparisons; Cohen’s d = 0.201 for public schools and 0.359 for private schools; Table 3).

### Predictors of participants’ BMI

In order to address potential confounding effects, two analytical models were used to evaluate factors influencing BMI (Table 4). Model 1 incorporated school type, gender, age (in years), PA level, and mean SB score. Model 2 included PA level but replaced the mean SB score with the scores for individual screen-based SBs: watching TV, laptop use, playing video games, and smartphone use. The *R*^2^ was 0.14 for Model 1 and 0.145 for Model 2, indicating that both models explained only a small portion of the variance in BMI.

**Table 4:** Parameter estimates for predictors of participants’ BMI.

Parameter	B	Std. Error	95% CI	Wald χ^2^	*p*	ES
Model 1						
(Intercept)	18.133	0.327	[17.491, 18.775]	3066.725	<0.001	N/A
[School = public]	0.081	0.054	[−0.024, 0.187]	2.269	0.132	0.037
[School = private]	0^a^					
[Gender = male]	0.492	0.056	[0.383, 0.601]	78.309	<0.001	0.226
[Gender = female]	0^a^					
Age	0.083	0.018	[0.048, 0.119]	21.149	<0.001	0.029
SB score	0.352	0.031	[0.291, 0.412]	131.521	<0.001	0.070
PA level	−0.626	0.038	[−0.700, −0.552]	275.347	<0.001	−0.104
[PA]^3^	−0.014	0.0067	[−0.027, −001]	4.750	0.029	−0.04
(Scale)	1.769^b^	0.047	[1.679, 1.864]			
Model 2						
(Intercept)	19.883	0.339	[19.219, 20.547]	3445.922	<0.001	N/A
[School = public]	0.074	0.054	[−0.032, 0.180]	1.891	0.169	0.034
[School = private]	0^a^					
[Gender = male]	0.553	0.064	[0.428, 0.678]	75.603	<0.001	0.254
[Gender = female]	0^a^					
Age	0.079	0.020	[0.041, 0.118]	16.621	<0.001	0.027
PA level	−0.633	0.038	[−0.708, −0.558]	274.164	<0.001	−0.106
[PA]^3^	−0.016	0.0067	[−0.029, −003]	5.781	0.016	−0.045
Type of SB score						
[Watching TV = Not at all]	−0.385	0.140	[−0.659, −0.110]	7.558	0.006	−0.177
[Watching TV = <30 min/day]	−0.462	0.104	[−0.666, −0.258]	19.729	<0.001	−0.213
[Watching TV = 30 min–1 h/day]	−0.448	0.088	[−0.620, −0.275]	25.874	<0.001	−0.206
[Watching TV = 1–2 h/day]	−0.219	0.079	[−0.373, −0.065]	7.773	0.005	−0.101
[Watching TV = 2–4 h/day]	−0.175	0.088	[−0.348, −0.002]	3.925	0.048	−0.081
[Watching TV = >4 h/day]	0^a^					
[Laptop use = Not at all]	−0.541	0.112	[−0.760, −0.323]	23.555	<0.001	−0.249
[Laptop use = <30 min/day]	−0.233	0.089	[−0.407, −0.058]	6.842	0.009	−0.107
[Laptop use = 30 min–1 h/day]	−0.127	0.089	[−0.302, 0.049]	2.005	0.157	−0.058
[Laptop use = 1–2 h/day]	−0.091	0.087	[−0.262, 0.079]	1.103	0.294	−0.042
[Laptop use = 2–4 h/day]	−0.045	0.096	[−0.234, 0.143]	0.222	0.637	−0.021
[Laptop use = >4 h/day]	0^a^					
[Video games = Not at all]	−0.270	0.110	[−0.486, −0.054]	6.003	0.014	−0.124
[Video games = <30 min/day]	−0.258	0.103	[−0.460, −0.056]	6.287	0.012	−0.119
[Video games = 30 min–1 h/day]	−0.392	0.103	[−0.594, −0.190]	14.422	<0.001	−0.180
[Video games = 1–2 h/day]	−0.251	0.099	[−0.445, −0.056]	6.390	0.011	−0.115
[Video games = 2–4 h/day]	−0.013	0.105	[−0.220, 0.193]	0.016	0.898	−0.006
[Video games = >4 h/day]	0^a^					
[Smartphone use = Not at all]	−0.338	0.173	[−0.676, 0.001]	3.825	0.050	−0.155
[Smartphone use = <30 min/day]	−0.358	0.095	[−0.544, −0.173]	14.303	<0.001	−0.165
[Smartphone use = 30 min–1 h/day]	−0.205	0.083	[−0.367, −0.043]	6.162	0.013	−0.094
[Smartphone use = 1–2 h/day]	−0.207	0.072	[−0.348, −0.066]	8.330	0.004	−0.095
[Smartphone use = 2–4 h/day]	0.069	0.078	[−0.084, 0.222]	0.790	0.374	0.032
[Smartphone use = >4 h/day]	0^a^					
(Scale)	1.746b	0.047	[1.657, 1.840]			

CI, confidence interval; ES, effect size; PA, physical activity; SB, sedentary behavior.

a Set to zero because this parameter is redundant.

b Maximum likelihood estimate.

Model 1 reported deviance and Pearson’s χ^2^ of 4979.336, resulting in a ratio of 1.773, indicating adequate model fit with some modest over-dispersion. The log-likelihood of −4797.058 further confirmed the model’s overall fit. The information criteria—Akaike information criterion (AIC; 9608.115), AIC corrected for small sample sizes (AICC; 9608.155), and Bayesian information criterion (BIC; 9649.714)—demonstrate that the model achieves a good balance between fit and complexity. The Omnibus test yielded a significant likelihood ratio (*χ*^2^ = 459.683; *p <* 0.001), indicating that this model, which includes school type, gender, age, PA level, and mean SB score as predictors, significantly outperforms an intercept-only model in predicting BMI.

In Model 2, the deviance and Pearson’s *χ*^2^ decreased to 4916.044, with a degrees-of-freedom ratio of 1.762, indicating a satisfactory fit with minor over-dispersion. The log-likelihood was −4779.052, supporting the model’s adequate fit. It had lower AIC (9610.104) and BIC (9764.615) than Model 1, suggesting an improved balance between complexity and fit after incorporating the specific types of SBs. The Omnibus test revealed a significant likelihood ratio (*χ*^2^ = 495.694, *p <* 0.001), demonstrating that including these factors significantly improves the model’s explanatory power.

The models demonstrated significant correlations between BMI and key predictors such as gender, age, PA level, and mean SB score. However, no significant relationship was observed between BMI and school type. Males exhibited a higher likelihood of elevated BMI than females, as evidenced by a positive *β* coefficient in Model 1 (*B* = 0.492; *p <* 0.001; ES = 0.226) and Model 2 (*B* = 0.553; *p <* 0.001; ES = 0.254). Age was positively associated with BMI in Model 1 (*B* = 0.083; *p <* 0.001; ES = 0.029) and Model 2 (*B* = 0.079; *p <* 0.001; ES = 0.027). The mean SB score was also positively associated with BMI in Model 1 (*B* = 0.352; *p <* 0.001; ES = 0.07), suggesting that BMI increases by 0.352 kg/m^2^ for each one-point increase in the mean SB score. In contrast, PA was negatively associated with BMI in Model 1 (*B* = −0.626; *p <* 0.001; ES = −0.104) and Model 2 (*B* = −0.633; *p <* 0.001; ES = −0.106).

The breakdown of SBs in Model 2 revealed distinct impacts. Watching TV was strongly associated with BMI, where participants watching TV for <4 h daily had lower BMIs than those watching TV for >4 h daily (the reference category), with *β* values from −0.175 to −0.462. Laptop use was more strongly associated with BMI, with a *β* of −0.541 for non-users (*p <* 0.001; ES = −0.249) and −0.233 for those using laptops for <30 min daily (*p* = 0.009; ES = −0.213); however, laptop use for more than 30 min daily did not significantly affect BMI. BMIs were lower among participants playing video games for up to 2 h daily than among those playing video games for >4 h daily (the reference category), with *β* from −0.251 to −0.392. Smartphone use was also negatively associated with BMI, particularly for non-users (*β* = −0.338; *p* = 0.050; ES = −0.155) or those who used them for <30 min (*β* = −0.358; *p <* 0.001; ES = −0.165), with smartphone use for 2–4 h not significantly affecting BMI.

### Predictors of participants’ BMI by gender

A polynomial regression analysis was conducted to examine possible non-linear relationships between BMI and age, PA level, and SB score. The analysis revealed a significant cubic relationship between PA level and BMI among males (*β*₁ = 1.264, *β*₂ = −1.029, *β*₃ = 0.167, *p <* 0.001) and females (*β*₁ = −3.406, *β*₂ = 1.595, *β*₃ = −0.276, *p <* 0.001). The *R*^2^ values were 0.080 and 0.086 for males in Models 1 and 2, respectively, and 0.123 and 0.130 for females. These findings indicate that the models explain only a small portion of the variance in BMI.

In males (Table 5), both models demonstrated significant likelihood ratios (Model 1: *χ*^2^ = 200.993, *p <* 0.001; Model 2: *χ*^2^ = 262.339, *p <* 0.001), indicating that the models fit significantly better than an intercept-only model. Comparisons of the information criteria (AIC and BIC) suggested that Model 2, which incorporated specific types of screen-based SBs, provided a better fit for explaining BMI variation. The generalized linear models assessing the effects of school type, age, PA level, and SB score on BMI—considering a cubic relationship—revealed significant predictors. In Model 1, PA level (*β* = −1.002; *p <* 0.001; ES = −0.14), age (*β* = 0.081; *p <* 0.001; ES = 0.025), and SB score (*β* = 0.381; *p <* 0.001; ES = 0.065) significantly affected BMI. The cubic term for PA level (PA^3^; *β* = 0.024; *p* = 0.005; ES = −0.061) indicated a non-linear relationship between PA levels and BMI. This finding suggests that the initial negative association between PA levels and BMI becomes more complex, with diminishing returns at higher PA levels. Specifically, while early increases in PA level are associated with decreases in BMI, this effect slows and can even reverse slightly at higher PA levels.

**Table 5:** Parameter estimates for BMI predictors among males.

Parameter	*B*	Std. Error	95% CI	Wald χ^2^	*p*	*ES*
Model 1						
(Intercept)	19.158	0.478	[18.221, 20.094]	1607.389	<0.001	N/A
[School = public]	−0.015	0.072	[−0.157, 0.127]	0.042	0.837	−0.007
[School = private]	0^a^					
Age	0.081	0.022	[0.038, 0.123]	13.901	<0.001	0.025
SB score	1.002	0.178	[−1.351, −0.652]	31.601	<0.001	0.17
PA level	−0.381	0.048	[0.286, 0.475]	62.453	<0.001	−0.053
[PA]^3^	−0.024	0.009	[0.007, 0.041]	7.839	0.005	−0.061
(Scale)	1.585b	0.058	[1.476, 1.702]			
Model 2						
(Intercept)	21.032	0.469	[20.113, 21.951]	2010.749	<0.001	N/A
[School = public]	−0.018	0.072	[−0.158, 0.122]	0.062	0.804	−0.008
[School = private]	0^a^					
Age	0.065	0.023	[0.020, 0.111]	7.952	0.005	0.019
PA level	−0.970	0.178	[−1.319, −0.622]	29.824	<0.001	−0.134
[PA]^3^	−0.022	0.009	[0.005, 0.039]	6.739	0.009	−0.055
*Types of SB score*						
[Watching TV = Not at all]	0.027	0.188	[−0.341, 0.395]	0.021	0.885	0.012
[Watching TV = <30 min/day]	−0.237	0.120	[−0.473, −0.001]	3.885	0.049	−0.109
[Watching TV = 30 min–1 h/day]	−0.282	0.115	[−0.506, −0.057]	6.046	0.014	−0.130
[Watching TV = >1–2 h/day]	0.113	0.112	[−0.106, 0.332]	1.027	0.311	0.052
[Watching TV = >2–4 h/day]	0.003	0.118	[−0.228, 0.234]	0.001	0.979	0.001
[Watching TV = >4 h/day]	0^a^					
[Laptop use = Not at all]	−0.303	0.177	[−0.650, 0.044]	2.926	0.087	−0.139
[Laptop use = <30 min/day]	−0.375	0.115	[−0.600, −0.151]	10.733	0.001	−0.172
[Laptop use = 30 min–1 h/day]	−0.333	0.113	[−0.554, −0.111]	8.673	0.003	−0.153
[Laptop use = >1–2 h/day]	−0.320	0.115	[−0.545, −0.095]	7.738	0.005	−0.147
[Laptop use = >2–4 h/day]	−0.077	0.125	[−0.322, 0.169]	0.376	0.540	−0.035
[Laptop use = >4 h/day]	0^a^					
[Video games = Not at all]	−0.484	0.169	[−0.815, −0.154]	8.266	0.004	−0.223
[Video games = <30 min/day]	−0.290	0.119	[−0.523, −0.058]	6.010	0.014	−0.133
[Video games = 30 min–1 h/day]	−0.525	0.112	[−0.744, −0.307]	22.198	<0.001	−0.241
[Video games = >1–2 h/day]	−0.275	0.105	[−0.482, −0.068]	6.805	0.009	−0.126
[Video games = >2–4 h/day]	0.029	0.107	[−0.181, 0.239]	0.073	0.787	0.013
[Video games = >4 h/day]	0^a^					
[Smartphone use = Not at all]	−0.108	0.248	[−0.593, 0.377]	0.191	0.662	−0.050
[Smartphone use = <30 min/day]	−0.353	0.108	[−0.566, −0.141]	10.629	0.001	−0.162
[Smartphone use = 30 min–1 h/day]	0.067	0.102	[−0.134, 0.267]	0.426	0.514	0.031
[Smartphone use = >1–2 h/day]	−0.157	0.094	[−0.342, 0.028]	2.755	0.097	−0.072
[Smartphone use = >2–4 h/day]	0.063	0.106	[−0.145, 0.271]	0.351	0.554	0.029
[Smartphone use = >4 h/day]	0^a^					
(Scale)	1.522^b^	0.055	[1.417, 1.635]			

CI, confidence interval; ES, effect size; PA, physical activity; SB, sedentary behavior.

a Set to zero because this parameter is redundant.

b Maximum likelihood estimate.

In Model 2, the cubic effect of PA level was further supported, with significant effects of age (*β* = 0.065; *p* = 0.005; ES = 0.019), PA level (*β* = −0.970; *p <* 0.001; ES = −0.134), and PA^3^ (*β* = 0.022; *p* = 0.009; ES = −0.055) on BMI. Additionally, specific types of SBs had notable effects on BMI. However, school type (public or private) did not significantly affect BMI.

In females (Table 6), Model 1 exhibited a deviance per degree of freedom (df) of 1.978, a log-likelihood of −2293.886, an AIC of 4601.771, and a BIC of 4637.989, indicating an adequate fit (*χ*^2^ = 237.192, df = 5, *p <* 0.001). Model 2 showed a slight improvement, with a deviance per df of 1.925, log-likelihood of −2266.278, AIC of 4584.557, and BIC of 4719.080 (*χ*^2^ = 292.406, df = 24, *p <* 0.001), suggesting that specific SBs better explain variation in BMI than the mean SB score.

**Table 6:** Parameter estimates for BMI predictors among females.

Parameter	*B*	Std. Error	95% CI	Wald χ^2^	*p*	ES
Model 1						
(Intercept)	16.422	0.6312	[15.185, 17.659]	676.863	<0.001	N/A
[School = public]	0.238	0.0833	[0.074, 0.401]	8.136	0.004	0.109
[School = private]	0^a^					
Age	0.104	0.0319	[0.041, 0.166]	10.577	0.001	0.043
SB score	0.436	0.0548	[0.329, 0.544]	63.453	<0.001	0.126
PA level	−0.211	0.1778	[−0.137, 0.560]	1.414	0.234	−0.046
[PA]^3^	−0.055	0.0103	[−0.076, −0.035]	28.670	<0.001	−0.230
(Scale)	1.969b	0.0771	[1.824, 2.126]			
Model 2						
(Intercept)	18.628	0.6794	[17.296, 19.959]	751.842	<0.001	N/A
[School = public]	0.209	0.0828	[0.047, 0.371]	6.373	0.012	0.096
[School = private]	0^a^					
Age	0.103	0.0350	[0.035, 0.172]	8.714	0.003	0.042
PA level	−0.205	0.1771	[−0.142, 0.552]	1.342	0.247	−0.044
[PA]^3^	−0.057	0.0103	[−0.078, −0.037]	31.434	<0.001	−0.250
*Types of SB*						
[Watching TV = Not at all]	−0.864	0.2077	[−1.271, −0.457]	17.290	<0.001	−0.397
[Watching TV = <30 min/day]	−0.656	0.2553	[−1.157, −0.156]	6.604	0.010	−0.302
[Watching TV = 30 min–1 h/day]	−0.577	0.1355	[−0.843, −0.311]	18.138	<0.001	−0.265
[Watching TV = >1–2 h/day]	−0.460	0.1101	[−0.676, −0.244]	17.443	<0.001	−0.212
[Watching TV = >2–4 h/day]	−0.360	0.1317	[−0.618, −0.101]	7.449	0.006	−0.166
[Watching TV = >4 h/day]	0^a^					
[Laptop use = Not at all]	−0.648	0.1493	[−0.940, −0.355]	18.823	<0.001	−0.298
[Laptop use = <30 min/day]	−0.166	0.1400	[−0.441, 0.108]	1.414	0.234	−0.076
[Laptop use = 30 min–1 h/day]	0.086	0.1468	[−0.202, 0.374]	0.343	0.558	0.040
[Laptop use = >1–2 h/day]	0.187	0.1335	[−0.075, 0.448]	1.955	0.162	0.086
[Laptop use = >2–4 h/day]	−0.018	0.1474	[−0.307, 0.270]	0.016	0.901	−0.008
[Laptop use = >4 h/day]	0^a^					
[Video games = Not at all]	−0.309	0.2605	[−0.820, 0.201]	1.409	0.235	−0.142
[Video games = <30 min/day]	−0.389	0.2657	[−0.910, 0.132]	2.144	0.143	−0.179
[Video games = 30 min–1 h/day]	−0.487	0.2709	[−1.018, 0.044]	3.235	0.072	−0.224
[Video games = >1–2 h/day]	−0.426	0.2697	[−0.954, 0.103]	2.488	0.115	−0.196
[Video games = >2–4 h/day]	−0.423	0.2986	[−1.009, 0.162]	2.010	0.156	−0.195
[Video games = >4 h/day]	0^a^					
[Smartphone use = Not at all]	−0.384	0.2444	[−0.863, 0.095]	2.469	0.116	−0.177
[Smartphone use = <30 min/day]	−0.125	0.1981	[−0.513, 0.264]	0.396	0.529	−0.057
[Smartphone use = 30 min–1 h/day]	−0.684	0.1371	[−0.953, −0.415]	24.889	0	−0.315
[Smartphone use = >1–2 h/day]	−0.269	0.1090	[−0.483, −0.056]	6.111	0.013	−0.124
[Smartphone use = >2–4 h/day]	0.057	0.1132	[−0.165, 0.279]	0.255	0.614	0.026
[Smartphone use = >4 h/day]	0^a^					
(Scale)	1.888^b^	0.0739	[1.748, 2.038]			

CI, confidence interval; ES, effect size; PA, physical activity; SB, sedentary behavior.

a Set to zero because this parameter is redundant.

b Maximum likelihood estimate.

Like in males, females’ BMI was affected by age (*β* = 0.104; *p* = 0.001; ES = 0.043) and SB score (*β* = 0.436; *p <* 0.001; *ES =* 0.126), indicating that greater SB is associated with greater BMI. The PA^3^ term (*β* = −0.055; *p <* 0.001; *ES = −*0.23) indicated a non-linear relationship, where BMI initially increases with PA level before decreasing at higher PA levels. However, unlike in males, attending a public school was associated with a higher BMI than attending a private school (*β* = 0.238; *p* = 0.004; *ES =* 0.109), and PA level showed no significant linear effect (*p* = 0.234).

In Model 2, which replaced the SB score with specific types of SBs (watching TV, laptop use, playing video games, and smartphone use), attending a public school was again associated with a higher BMI than attending a private school (*β* = 0.209; *p* = 0.012; *ES =* 0.096), PA level showed no significant linear effect (*p* = 0.247), and the PA^3^ term remained significant and negative (*β* = −0.057; *p <* 0.001; *ES = −*0.25). Age was positively associated with BMI (*β* = 0.103; *p* = 0.003; *ES =* 0.042). Watching TV showed significant negative associations with BMI, with *β* from −0.864 (*p <* 0.001; *ES = −*0.397) for non-viewers to −0.360 (*p* = 0.006; *ES = −*0.166) for those watching TV for 2–4 h daily. Non-users of laptops had significantly lower BMI (*β* = −0.648; *p <* 0.001; *ES = −*0.298), while laptop use did not significantly affect BMI. Moderate smartphone use (<30 min/day and 30 min–1 h/day) was associated with lower BMI (*β* = −0.684; *p <* 0.001; *ES = −*0.315) and (*β* = −0.269; *p* = 0.013; *ES = −*0.124), respectively, while longer smartphone use did not significantly affect BMI. Playing video games generally had a negligible impact on BMI.

## Discussion

Our study revealed patterns in BMI, obesity, PA level, and SB score among adolescents in the Al-Ahsa Region of Saudi Arabia, including variations by school type (public vs. private) and gender. It also identified key factors affecting BMI, examining the influences of school type, gender, age, PA level, and general and specific SBs, including watching TV, laptop use, playing video games, and smartphone use.

### Prevalence of OW and OB

Our findings revealed that 28.4% of participants were classified as OW or OB, with 17.2% categorized as OB. Among males, 41.1% were OW or OB, with 26.9% classified as OB, whereas among females, only 14.9% were OW or OB, with 6.0% classified as OB. A comparison of school types showed that the prevalence of OW was lower among private school students than public school students (9.9% vs. 11.8%), while the prevalence of OB was nearly identical (17.3% vs. 17.2%).

This study indicates a slightly higher prevalence of OW than previous national studies. For example, Alhamed et al. (46) analyzed data from a cross-sectional study of 1,134,317 Saudi children and adolescents as part of the Ministry of Health’s school screening program. They reported an overall prevalence of OW (10.4%) and OB (10.7%), with prevalences higher among males (OW = 10.8%, OB = 12.5%) than females (OW = 10.1%, OB = 9.1%). The prevalence of OW (12.6%) and OB (12.3%) was higher among intermediate school students than among primary and secondary school students. Similarly, a multicenter study by AlEnazi et al. (47) involving 91,676 adolescents aged 14–19 years found that 66.8% were NW, 10.9% were UW, 12.8% were OW, and 9.5% were OB. The prevalences of OW and OB were higher among males (12.2 and 10.5%) than females (10.3% and OB). In the Eastern Region, the prevalences of OW and OB were 12.4 and 10.2%, respectively.

In another study by Al-Hussaini et al. (48) involving 7,930 participants aged 6–16 years from Riyadh, the overall prevalence of OW and OB was 13.4% (14.2% in females vs. 12% in males) and 18.2% (18% in females vs. 18.4% in males), respectively. The prevalence of both OW and OB was higher among adolescents (14.6 and 20.2%) than children (12 and 15.7%). Interestingly, females were more likely to be OW than males (14.2% vs. 12%), while the prevalence of OB did not differ significantly by gender.

The prevalence of OB is notably higher among Saudi adults (aged ≥18 years) than adolescents. For example, Althumiri et al. (1) reported prevalences of OB in adults of 22.2% in 2020, 22.1% in 2021, 21.2% in 2022, and 21.4% in 2023, based on data from 92,137 participants representing all 13 administrative regions of Saudi Arabia. Unlike in adolescents, the prevalence of OB was consistently higher among adult females than males, with a prevalence of 24.4% in 2020 and 2021, decreasing slightly to 23.5% in 2022 and 23.4% in 2023.

When comparing Saudi to global adolescents, they occupy an intermediate position in OW and OB prevalence. For example, in the United States, 22.2% of adolescents aged 12–19 years were OB (49). In China, Cheng et al. (50) found that among 1,196,004 school-aged children, the prevalence of OW and OB was 11.8 and 8.1%, respectively. Similarly, Song et al. (51) reported a mean OB prevalence of 8.25% among 1,677,261 Chinese adolescents aged 7–18 years, with a UW prevalence of 3.39%. In Europe, the prevalence of OW and OB among 15-year-olds was 19% on average across EU countries in 2018, up from 16% in 2010, with prevalences ranging from 12% in the Netherlands to 36% in Malta (52). Across most EU countries, OW and OB are more common in males (23%) than females (15%) due to a combination of biological, social, and environmental factors.

In contrast, Al-Nuaim and Safi (20) observed no significant gender differences in the prevalences of OW or OB among 380 secondary school students (199 males, 181 females) from various regions of Saudi Arabia. They reported the prevalence of OB of 18.09% among males and 19.10% among females and a prevalence of OW of 27.07% among males and 11.60% among females. Similarly, Al-Nuaim et al. (53) found no significant gender differences in BMI among 1,270 Saudi youths aged 15–19 years, although they observed differences in waist circumference. Among males, 26.5% were classified as “at risk,” compared to 54.2% of females. Additionally, the proportion of “at-risk” individuals was higher among private school students (45.5%) than among public school students (38.6%).

### Determinants of BMI

Obesity among adolescents in Saudi Arabia arise from a complex interplay of biological, behavioral, social, and environmental factors, reflecting global trends (54). Our findings align with this framework, revealing significant gender and school-type disparities in PA and SB, which influence BMI. Consistent with previous research, our findings indicate notable gender disparities. Males generally exhibit higher PA levels compared to females; however, these benefits may be offset by engagement in SBs such as video gaming, often accompanied by high-calorie diets (55). In contrast, females tend to have lower PA levels and spend more time in SBs, influenced by societal norms and limited access to opportunities for vigorous PA (56, 57).

Educational settings play a crucial role in shaping PA opportunities. Our research shows higher overweight/obesity rates among public school students compared to private school students, which may be attributed to disparities in resources, nutrition programs, and access to physical education, particularly for female students (47). These findings align with previous studies that highlight the impact of economic and infrastructural differences on PA and SB patterns among adolescents in Saudi Arabia (16, 24). Unhealthy dietary habits and decreased PA are identified as significant factors contributing to the increasing rates of OW and OB in Saudi adolescents (58). Washi and Ageib (59), examining the dietary habits and quality of food among Saudi youth, revealed an increase in energy intake from fats. They noted that rice, bread, and meat are considered staple foods, incorporated into nearly every meal. This aligns with other studies focusing on this age group, which indicate that obese adolescents have higher consumption levels of meat, grain products, fast foods, sugary beverages, and potato chips. These factors result in a greater caloric intake in comparison to non-obese adolescents (60).

Rapid urbanization and economic growth have intensified these challenges by enhancing access to high-fat, calorically dense foods and encouraging sedentary lifestyles, influenced by dependence on motorized transportation and excessive screen time (48, 61). The combination of lifestyle changes and cultural and environmental barriers poses substantial challenges to PA engagement, especially among females (21, 24). The harsh climate, marked by intense summer heat and cold, windy winters, limits opportunities for PA for much of the year. The problem is exacerbated by the lack of exercise facilities at Al-Ahsa (53). Cultural attitudes and beliefs frequently diminish the value of PA as a leisure pursuit, especially in rural regions. Academic achievement is typically emphasized more than PAs, with parents often promoting involvement in educational and spiritual endeavors instead of PAs. The absence of public parks, sports facilities, and other venues conducive to youth PA restricts exercise opportunities in the Al-Ahsa region.

Finally, the analysis elucidates the relationship between national obesity trends and regional variances in BMI determinants, encompassing gender and school-type disparities. The findings highlight the necessity for focused interventions that rectify these discrepancies, including the establishment of supportive conditions for PA, enhancement of school-based health initiatives, and the resolution of cultural obstacles to female engagement in PA.

### Influence of school type

Unexpectedly, the school type did not greatly influence BMI, indicating that the school environment may have a weaker impact on BMI in adolescents in this cultural setting. Our findings demonstrated significant differences in overweight/obesity rates, PA levels, and SB scores between public and private schools. However, regression models indicated that school type only had a significant association with BMI among females. This finding aligns with analogous findings in Saudi Arabia, highlighting the impact of behavioral determinants on adolescent health outcomes (16). While the school type may influence other dimensions of adolescents, such as well-being and mental health (62), it does not appear to significantly affect BMI (53).

Various cultural and environmental factors may determine these outcomes. Religious rituals in Saudi Arabia enforce specific regulations that affect PA (63), while the severe Saharan climate—marked by extreme summer heat, strong winter winds, and frequent sandstorms—restricts opportunities for regular PA (64). Moreover, the absence of regulated PE classes, particularly in females’ schools, constitutes a substantial obstacle to fostering regular PA (22).

Nonetheless, our findings contradict research indicating that the school environment significantly affects PA and SB in adolescents. One reason for this contradiction could be that many interventions integrating health education with supplementary PA sessions often fail because they overlook broader environmental factors, such as institutional policies and societal norms (65). While socioeconomic improvements have enhanced access to resources and infrastructure in many areas, public schools still face limitations in terms of facilities and extracurricular programs. Moreover, although physical education classes have been scheduled for female students since 2014, the implementation of this policy was hindered by objections from conservatives who view it as immodest, and it is not mandatory (66).

Furthermore, the lack of sufficient outdoor facilities or safe spaces for PA—particularly during extreme weather conditions in Al-Hasa—can significantly affect both PA levels and SB scores. These environmental challenges, combined with limited access to structured programs for PA and sports, likely contribute to the absence of a significant relationship between BMI and PA/SB patterns in both public and private school students in this region (48, 53, 61).

The Health-Promoting Schools framework is a promising strategy that incorporates modifications to the school environment, curriculum-based health teaching, and community engagement to foster healthier behaviors. This strategy has shown promise in enhancing PA; nonetheless, information distinguishing the specific impact of environmental factors on PA is scarce, especially concerning adolescents. This observation highlights the need for focused research to enhance comprehension of how schools might maximize their contribution to fostering healthier habits (67).

### PA level, SB score, and BMI in males

Both of our models exhibited low *R*^2^ values with slight over-dispersion, suggesting that a substantial portion of the variance in BMI is not accounted for by the included predictors. The R^2^ values for Models 1 and 2 were 0.080 and 0.086, respectively, indicating that the models explain only a small portion of the variance in BMI. Therefore, additional, unmeasured factors, including dietary habits (68), genetic predisposition (69), and psychosocial factors (70), may significantly influence BMI. Our results indicate a complex relationship between BMI and variables such as PA, SB, age, and school type among Saudi adolescents, revealing significant differences in their effects on BMI. Age variability, particularly within a sample of 13–18 years, is likely to reflect growth and developmental changes that may lead to over-dispersion in the models. Adolescence is a period characterized by significant physical and hormonal transformations that might affect BMI, PA, and SB. This variability may partially account for the slight over-dispersion noted in the GLMs.

PA consistently serves as a protective factor against elevated BMI, with increased PA levels associated with lower BMI (71). This pattern highlights the need to encourage PA among Saudi adolescents to address increasing OB rates. The notable non-linear effects of PA indicate that both sustained and varied intensities of PA may provide cumulative benefits. This observation suggests that interventions should promote a range of PAs over time rather than concentrating exclusively on achieving minimum PA thresholds (72).

SBs exhibited specific effects, especially screen-based SBs. The inverse relationships observed between BMI and TV watching, laptop use, and smartphone use indicate that these types of SBs do not consistently correlate with elevated BMI and may, in specific contexts, be associated with lower BMI (73). The findings indicate that various forms of SB may not present uniform health risks, possibly attributable to compensatory PA or differing metabolic responses associated with specific SBs. Screen-based activities may correlate with physical movement breaks or reduced food consumption relative to other activities (74).

Male participants exhibited a greater tendency for elevated BMI than female participants, as evidenced by the positive coefficients for gender in both models. In males, BMI was significantly affected by SBs, including watching TV and smartphone use. Our data indicated that males who watched TV for <30 min daily had a 0.237 kg/m^2^ BMI than those who watched TV for >4 h daily. Similarly, those who used smartphones for <30 min daily had a 0.353 kg/m^2^ BMI. Our findings highlight the need to reduce screen time to manage BMI in male adolescents (73). School type did not significantly affect BMI, even though private schools generally provide more organized PE programs and extracurricular activities that may encourage healthier lifestyles. This observation indicates that behavioral factors, including PA and screen time, have a greater impact on BMI than the type of school attended (75, 76).

### PA level, SB score, and BMI in females

Our study underscores a similar pattern among females concerning the advantageous effects of heightened PA and the need to reduce SB. Nevertheless, Models 1 and 2 only explained a small part of the variation in BMI, with R^2^ values of 0.123 and 0.130, and a slight over-dispersion in the models. This might be due to the natural growth variability and developmental changes that happen during adolescence. The association between BMI and its predictors is clearly complex, given the significant physical and hormonal changes that girls go through between the ages of 13 and 18.

Nonetheless, our findings demonstrated that females seemed less impacted by the duration of SB than males, as the adverse correlation between SB and BMI exhibited lower variability across varying durations of watching TV, laptop use, and smartphone use. The substantial impact of school type on females’ BMI underscores the need for additional research. Female students at private schools exhibited lower BMIs and greater PA levels and SBs than those in public schools. This disparity may be attributed to differences in access to PE and extracurricular activities, as PE is not routinely provided in all Saudi schools (16). Private schools are more inclined to provide organized PE sessions and recreational activities, resulting in greater PA levels and lower BMIs among private school females. Nonetheless, their greater SB may indicate lifestyle characteristics linked to higher socioeconomic class, including enhanced access to technology for educational and recreational activities. These findings emphasize the dual function of school surroundings influencing PA and SB patterns and reinforce the need for nationwide initiatives to standardize PE programs across all schools (77). Future research should examine how school policies and tailored interventions that promote PA and reduce SB can alleviate BMI differences between public and private schools.

### Relation between PA level and BMI

Our findings indicate significant cubic relationships between PA and BMI in Saudi adolescents, aligning with prior studies that proposed non-linear associations between PA and BMI (78–80). In males, BMI generally remained constant at low and moderate PA levels but exhibited a modest increase at the highest PA levels. Conversely, females demonstrated a more significant non-linear trend: BMI first declined with increasing PA, steadied, and then slightly increased at high PA levels. These findings indicate diminishing returns, where initial increases in PA result in a decrease in BMI, while subsequent increases may yield no further advantages and could increase BMI (81).

The identified gender disparities may arise from differences in the intensity or type of PAs commonly undertaken by males and females, metabolic or hormonal differences, or dietary habits linked to differing PA levels (82). The increased diversity in males relative to females indicates a potential need for tailored interventions to address these differences. The notably lower BMI among males who spent less time doing screen-based activities highlights the robust correlation between SBs and BMI, emphasizing the need to restrict screen time to enhance weight outcomes (83, 84).

Our findings are consistent with recent research demonstrating non-linear relationships between PA and BMI in adolescents (85, 86), highlighting that while moderate PA is advantageous, extremely high PA may not consistently lead to additional decreases in BMI. Future research should explore the fundamental mechanisms driving these non-linear trends and assess other confounding factors, including dietary intake and socioeconomic status, that may affect these associations (87).

### Implications for public health

Our findings emphasize the need to promote PA and reduce SB as critical strategies to address the increasing prevalence of OW and OB among Saudi adolescents. The type of school does not significantly affect BMI; lifestyle factors such as screen-based SBs and PA levels are acknowledged as more crucial drivers. Policy recommendations include that interventions should focus on minimizing SB, encouraging varied and continuous PA, and addressing cultural obstacles that hinder females’ engagement in PA. To this end, the Saudi government must ensure that comprehensive physical education programs, particularly for females, are consistently implemented, while school administrators should prioritize investing in climate-resilient facilities such as indoor gyms. Public health specialists should collaborate with schools to provide culturally relevant initiatives that encourage a healthy diet and regular PA. Furthermore, community-based initiatives should provide structured opportunities for PA outside of school, especially in regions like Al-Hasa where environmental conditions limit outdoor activities.

The Health-Promoting Schools framework provides an efficient approach to integrating health education with environmental and regulatory changes to foster healthier behaviors, but its implementation requires coordination across multiple sectors to ensure sustained impact (16, 53, 61, 77).

### Limitations and future research

This study has some limitations. First, the low *R*^2^ values for both models suggest that a substantial portion of the variance in BMI remains unexplained, emphasizing the need for future research to incorporate additional predictors, such as dietary habits, genetic predispositions, and socioeconomic factors (54). External factors, including unmeasured variables, social determinants, and physiological or environmental influences, likely contribute to BMI outcomes and may explain the over-dispersion observed in both models. Secondly, the dependence on self-reported data presents possible biases, as individuals may inadvertently provide false evaluations of their behaviors due to memory constraints or deliberately provide good results. Employing direct and objective metrics of PA and SB, such as accelerometry or pedometry, would produce more accurate outcomes. Third, relying solely on BMI as an indicator of overweight/obesity status may restrict the comprehensive understanding of these conditions’ multifaceted character. Future studies incorporating additional metrics, such as PBF, waist circumference, or waist-to-hip ratio, could provide additional details that help better understanding factors influencing these outcomes. Finally, further research should explore how school policies and practices can be refined to more effectively influence BMI outcomes, especially for females, in order to develop more tailored and effective interventions (77).

## Conclusion

Our study revealed a notable increase in the prevalence of OW and OB among Saudi adolescents in the Al-Ahsa region, which were significantly higher in males than females. Among participants, 28.4% were identified as OW or OB, with 17.2% classified as OB. The prevalence was higher in males (41.1% OW/OB, including 26.9% OB) than in females (14.9% OW/OB, including 6.0% OB).

A comparison of school types indicated that the prevalence of OW was marginally lower in private schools (9.9%) than in public schools (11.8%), whereas the prevalence of OB was comparable in private schools (17.3%) and public schools (17.2%). Nonetheless, a significant relationship between BMI and school type was observed among females but not all participants or males.

Our analysis identified significant predictors of BMI, including gender, age, PA levels, and mean SB score. Males appeared at greater risk of increased BMI than females, as evidenced by a positive *β* coefficient in Model 1 (0.492) and Model 2 (0.553). The associations of age and mean SB scores with BMI were positive, while specific SBs exhibited differing effects. In contrast, PA exhibited a protective effect on BMI, as elevated PA levels correlated with lower BMI, with a B of −0.626 in Model 1 and −0.633 in Model 2.

The polynomial terms indicated a more suitable cubic relationship between BMI and PA among all participants, males, and females. The R2 was 0.140 for Model 1 and 0.145 for Model 2 among all participants, 0.080 and 0.086 among males, and 0.123 and 0.130 among females. These findings suggest that the models explain only a small portion of the variance in BMI. Future research should investigate the underlying mechanisms driving these non-linear trends and evaluate additional confounding factors, such as dietary intake and socioeconomic status, that may influence these associations (87).

## Data Availability

The raw data supporting the conclusions of this article will be made available by the authors, without undue reservation.
